# Role of microRNA-4516 involved autophagy associated with exposure to fine particulate matter

**DOI:** 10.18632/oncotarget.9978

**Published:** 2016-06-13

**Authors:** Xiaobo Li, Yang Lv, Jihong Hao, Hao Sun, Na Gao, Chengcheng Zhang, Runze Lu, Shizhi Wang, Lihong Yin, Yuepu Pu, Rui Chen

**Affiliations:** ^1^ Key Laboratory of Environmental Medicine Engineering, Ministry of Education, School of Public Health, Southeast University, Nanjing 210009, China; ^2^ Department of Histology and Embryology, Hebei North University, Zhangjiakou 075000, China; ^3^ Clinical Laboratory of The Second Hospital, Hebei Medical University, Shijiazhuang 050000, China; ^4^ State Key Laboratory of Bioelectronics, Southeast University, Nanjing 210096, China

**Keywords:** PM_2.5_, metal, autophagy, ribosome, microRNA

## Abstract

Metals are vital toxic components of fine particulate matter (PM_2.5_). Cellular responses to exposure to PM_2.5_ or PM metal components remain unknown. Post-transcriptional profiling and subsequent cell- and individual-based assays implied that the metal ion-binding miR-4516/RPL37/autophagy pathway could play a critical role in cellular responses to PM_2.5_ and PM metal stresses. miR-4516 was up-regulated in A549 cells exposed to PM_2.5_ and in the serum of individuals living in a city with moderate air pollution. The expression levels of the miR-4516 target genes, namely, RPL37 and UBA52, were involved in ribosome function and inhibited by exposure to PM_2.5_ and PM metal components. Autophagy in A549 cells was induced by PM_2.5_ exposure as a response to decreased RPL37 expression. Moreover, enhanced miR-4516 expression was positively correlated with the augmentation of the internal burden of aluminum and lead in individuals living in a city with moderate air pollution. Hereby, the miR-4516/RPL37/autophagy pathway may represent a novel mechanism that mediates responses to PM metal components.

## INTRODUCTION

Accumulated evidence from epidemiological studies supports the association between fine particulate matter (PM_2.5_) and increased risk of respiratory damages worldwide [1–3]. Outdoor air pollution and its PM were classified as carcinogenic to humans by the International Agency for Research on Cancer in 2013 [4]. PM_2.5_ is a mixture of multiple particles from various origins, varying with locations, and is chemically unspecific [5]. In previous studies, adverse effects were observed even when PM concentration complied according to the criteria or within “acceptable” levels [6, 7], which suggested that PM mass concentrations alone might not account for the entire health outcomes. Therefore, in addition to mass concentration, the chemical composition of PM_2.5_ could play a role in inducing harmful effects [8, 9].

Both heavy and transition metals, such as lead (Pb), arsenic (As), zinc (Zn), aluminum (Al), titanium (Ti), manganese (Mn), copper (Cu), nickel (Ni), cadmium (Cd), and iron (Fe), are major toxic components of PM_2.5_ [7, 10–13]. These elements are associated with adverse health effects. Exposure to PM Al, Ni, and Ti can increase the risk of low birth weight, despite that the exposure levels are in compliance with the corresponding air pollution standards [7]. High metal burden within the same cerebral region induces neuroinflammation and DNA damage in olfactory bulbs of children and young adults [14]. Metal components of PM are the main contributors to air pollution-related enhancement of hospital admissions and lifetime risk of death from cardiovascular diseases [15]. Pb, Cd, As, and Cr are associated with Pb-containing ores. Therefore, Pb emitted from various industrial processes is usually accompanied with these toxic metals. Heath risks induced by Pb exposure of occupational workers or children have been widely investigated [16, 17]. Pb exposure was found to be positively correlated with serum levels of miR-222; hence, microRNA (miRNA) expression might represent a vital mechanism to mediate individual responses to PM metal components [18].

miRNAs are endogenous small-noncoding RNA molecules with length of 20 to 22 nucleotides [19]. Dysregulation of miRNAs is involved in various biological and pathological processes [20] because of their interactions with a broad set of human genes. PM, carbon black particles, and PM metals can alter miRNA exposure *in vitro* and *in vivo* [18, 21–24]. Upon reduction in their target gene expression, circulating miRNAs could be used as potential biomarkers to reflect correlations with environmental stresses [25]. Maternal and cord blood miR-223 levels are correlated with maternal urine cotinine levels and low immune regulation induced by tobacco smoking [26].

In this study, we hypothesized that modulated microRNA expression could be a stress response to exposure to PM_2.5_ or PM metals and are involved in alterations in cellular phenotypes. The interaction of miRNA potential target genes and cellular responses were evaluated to clarify the underlying mechanisms activated by exposure to PM metals.

## RESULTS

### miR-4516 expression was validated in A549 cells and individual serum

**Figure 1:**
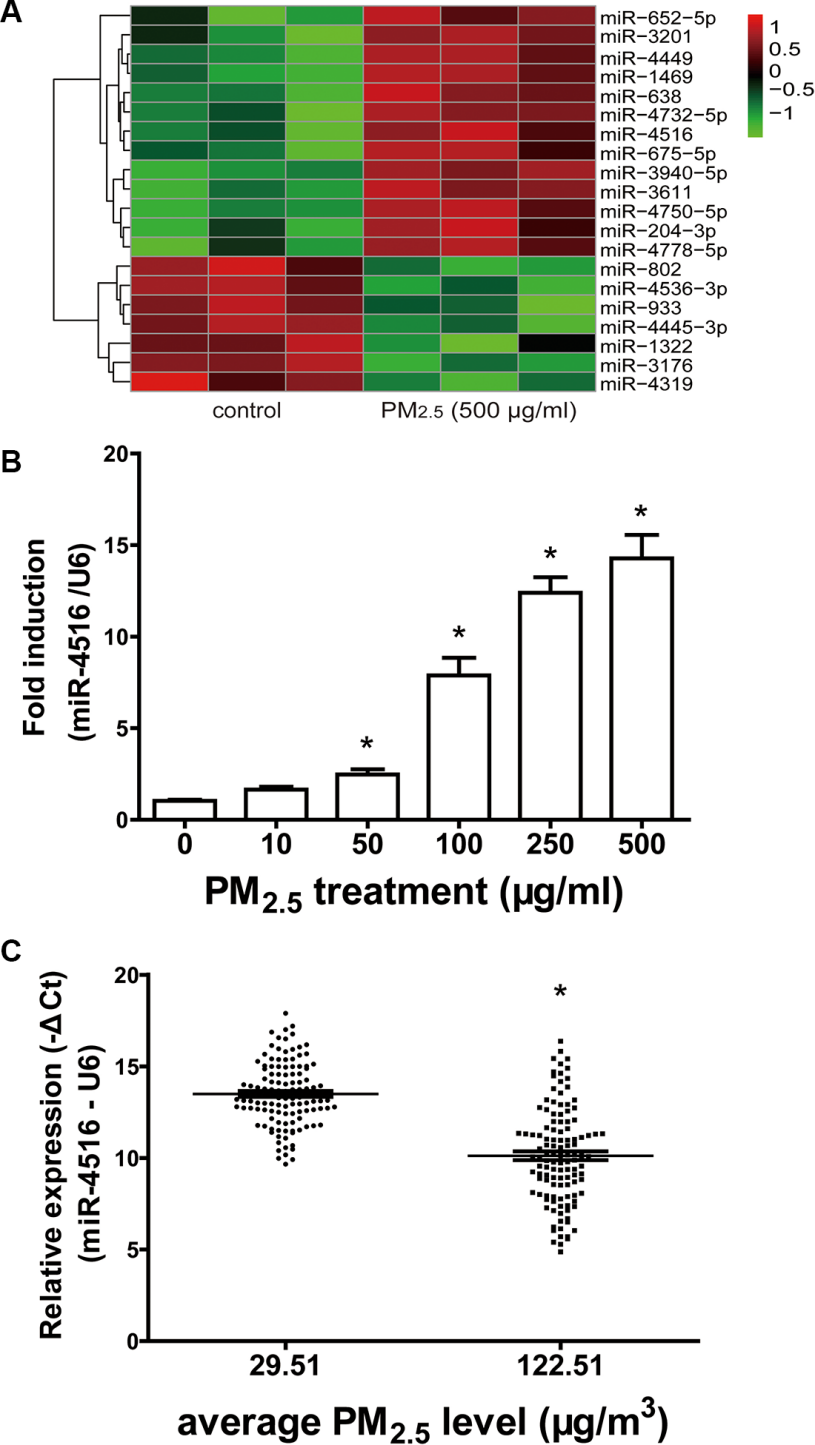
Modulation of miR-4516 expression in A549 cells and individual serum (**A**) The heatmap showed the induced and suppressed miRNAs of samples exposed to 500 μg/ml PM_2.5_ relative to matched controls in terms of the fold-change values as 2.0, *P* = 0.05. (**B**) Enhanced expression of miR-4516 in A549 cells treated with PM_2.5_ in a dose-dependent manner. *P < 0.05, compared with control. (**C**) miR-4516 levels were significantly increased in the serum of residents living in a moderately polluted city. *P < 0.05, compared with city with clean air.

Microarray profiling in A549 cells showed significantly modulated miRNAs. Thirteen up-regulated and seven down-regulated miRNAs were identified in A549 cells treated with 500 μg/mL PM_2.5_ for 24 h, with a cut-off fold change (FC) of 2 or more (*P* < 0.05, Table S1). The heatmap presented the induced and suppressed miRNAs of samples exposed to 500 μg/mL PM_2.5_ relative to the matched controls (Figure 1A). miR-4516 was the most modulated miRNA, with an FC of 9.687 and FDR of 0.0136. miR-4516 expression in A549 cells and sera was confirmed by qRT-PCR analysis. The expression of miR- 4516 was enhanced in A549 cells exposed to PM_2.5_ in a dose-dependent manner and significantly increased in 50, 100, 250, and 500 μg/mL PM_2.5_-treated groups (Figure 1B). Moreover, miR-4516 expression was significantly augmented in the serum of residents living in a moderately polluted city than those from a city with good air quality (Figure 1C). miR-4516 expression was not significantly correlated with age or sex characteristics (Table S2).

### Bioinformatics analysis for exploring the critical pathway of miR-4516 target genes

**Figure 2:**
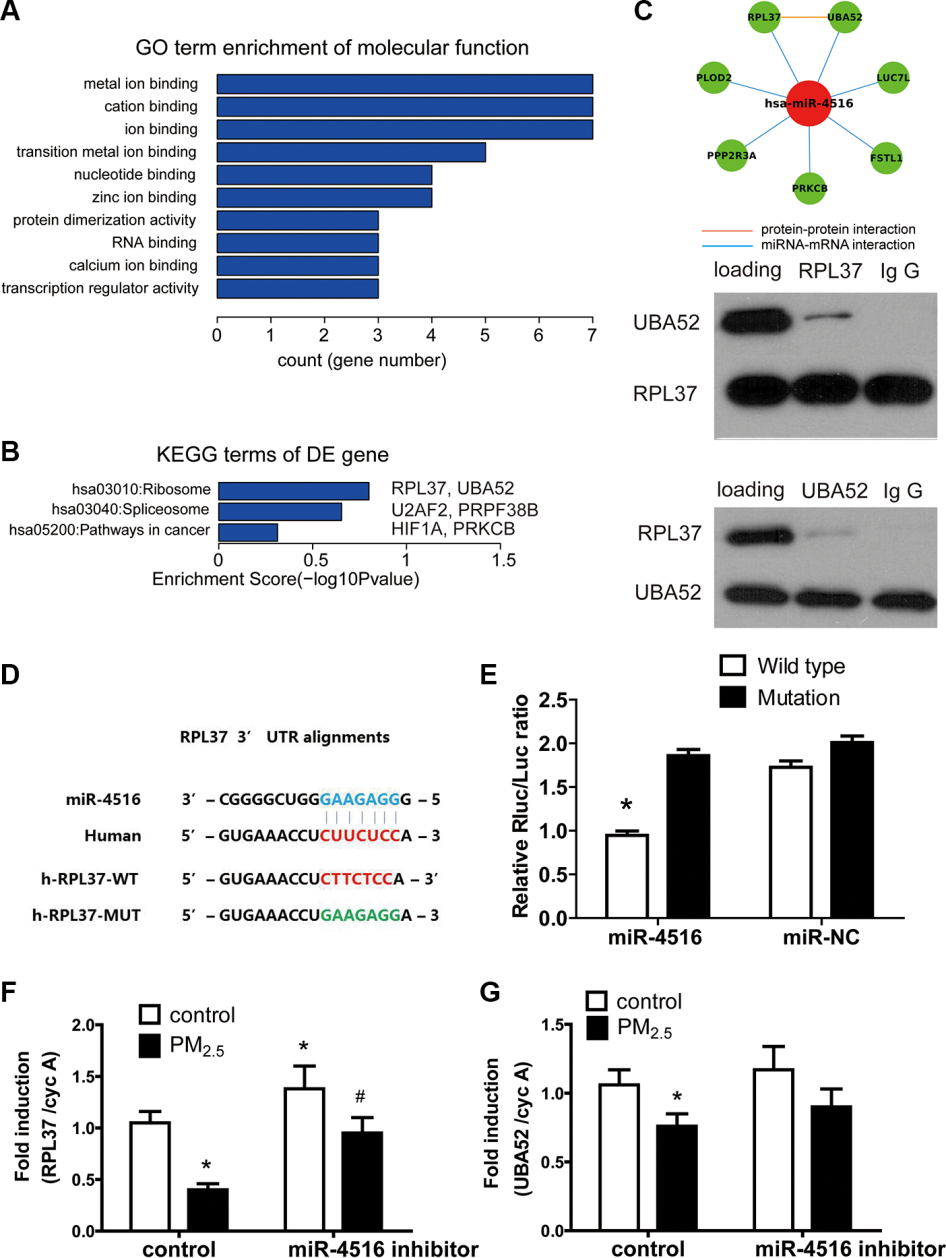
Functional annotations of miR-4516 targets A total of 25 targets of miR-4516 were determined by target prediction coupled with proteomics data. (**A**) Enriched GO terms of the 25 targets are shown by bar plot. Enrichment score of each term represents the term involved gene numbers. (**B**) UBA52 and RPL37 were enriched in the KEGG term “ribosome”. (**C**) The interaction between RPL37 and UBA52 was revealed by PPI analysis and confirmed by CO-IP assays. (**D**) The potential biding site of miR-5416 to RPL37 3′-UTR was predicted by the Targetscan online tool. The corresponding mutated sites of RPL3 3′-UTR were labeled in green. (**E**) The luciferase reporter assay showed that miR-4516 mimics the significantly decreased luciferase activity in 3′-UTR of RPL37. *P < 0.05, compared with the other three groups. (**F**) The expression of RPL37 in A549 cells was significantly decreased by treatment with 100 μg/ml PM_2.5_ for 24 h and increased after miR-4516 inhibitor transfection for 48 h. Co-treatment of PM_2.5_ and the miR-4516 inhibitor rescued RPL37 expression to control levels. (**G**) Treatment with 100 μg/ml PM_2.5_ significantly decreased UBA52 expression in A549 cells. *P < 0.05, compared with untreated control, ^#^*P* < 0.05, compared with control within each group.

miRNAs exert biological functions via modulation of their target genes. To determine the biological role of miR-4516 in A549 cells, we explored the targets of miR-4516 by comparing predicted target mRNA miRWalk database [27] with the entire proteomics data. LC-MS/MS-based protein profiling was employed to comprehensively determine the modulated proteins in A549 cells. Twenty-five protein-encoding mRNAs (Table S3), which are potential miR-4516 targets, were downregulated in proteomics profiling (FC > 1.5) and analyzed by GO and KEGG. Results showed that the significantly enriched term for sub-ontologies of molecular function was related to metal ion-binding (Figure 2A and Table S4). Enrichments for sub-ontologies of biological processes and cellular component are shown in Tables S5 and S6. Seven genes including PPP2R3A, PLOD2, RPL37, FSTL1, LUC7L, UBA52, and PRKCB are involved in metal ion-binding processes. Furthermore, UBA52 and RPL37 are related to ribosome pathway, and PRKC is related to the “cancer” pathway, as indicated by the KEGG analysis (Figure 2B). Protein–protein interaction (PPI) analysis demonstrated potential physical interaction between RPL37 and UBA52 (Figure 2C). Subsequently, the CO-IP assay was performed to explore the interaction between these two proteins. As shown in Figure 2C, the binding of RPL37 and UBA52 was observed when the extracted protein was incubated with the primary antibody of RPL37. When incubated with the primary antibody of UBA52, intensive expression of UBA52 and slight expression of RPL37 were observed in A549 cells. The results confirmed the interaction between RPL37 and UBA52, and the former plays a predominant role in this interaction.

Luciferase reporter assay was used to validate the regulation of miR-4516 to RPL37. Figure 2D shows the sequence of wild type RPL37 and mutated RPL37 3′-UTR sequence. The luciferase signals of A549, which was transfected with different reporter gene plasmids, are shown in Figure 2E. Co-transfection of miR-4516 with wild-type plasmid showed significant inhibition of luciferase activities in A549 cells as compared with the mutants.

A549 cells were treated with 100 μg/mL PM_2.5_, with or without the miR-4516 inhibitor to explore the role of miR-4516 in gene expression decreasing induced by PM_2.5_ exposure. As shown in Figures 2F and 2G, RPL37 and UBA52 mRNA expression levels were inhibited after 100 μg/mL PM_2.5_ treatment for 24 h, which was already demonstrated in previous studies. The expression level of RPL37 increased after transfection of the miR-4516 inhibitor for 48 h. Co-treatment of the miR-4516 inhibitor with PM_2.5_ could restore RPL37 and UBA52 mRNA expression to control levels, suggesting the miR-4516 based suppression of PM_2.5_ to RPL37 and UBA52 expression.

### Expression of RPL37 and UBA52 was regulated by PM_2.5_ and their major metal components

**Figure 3:**
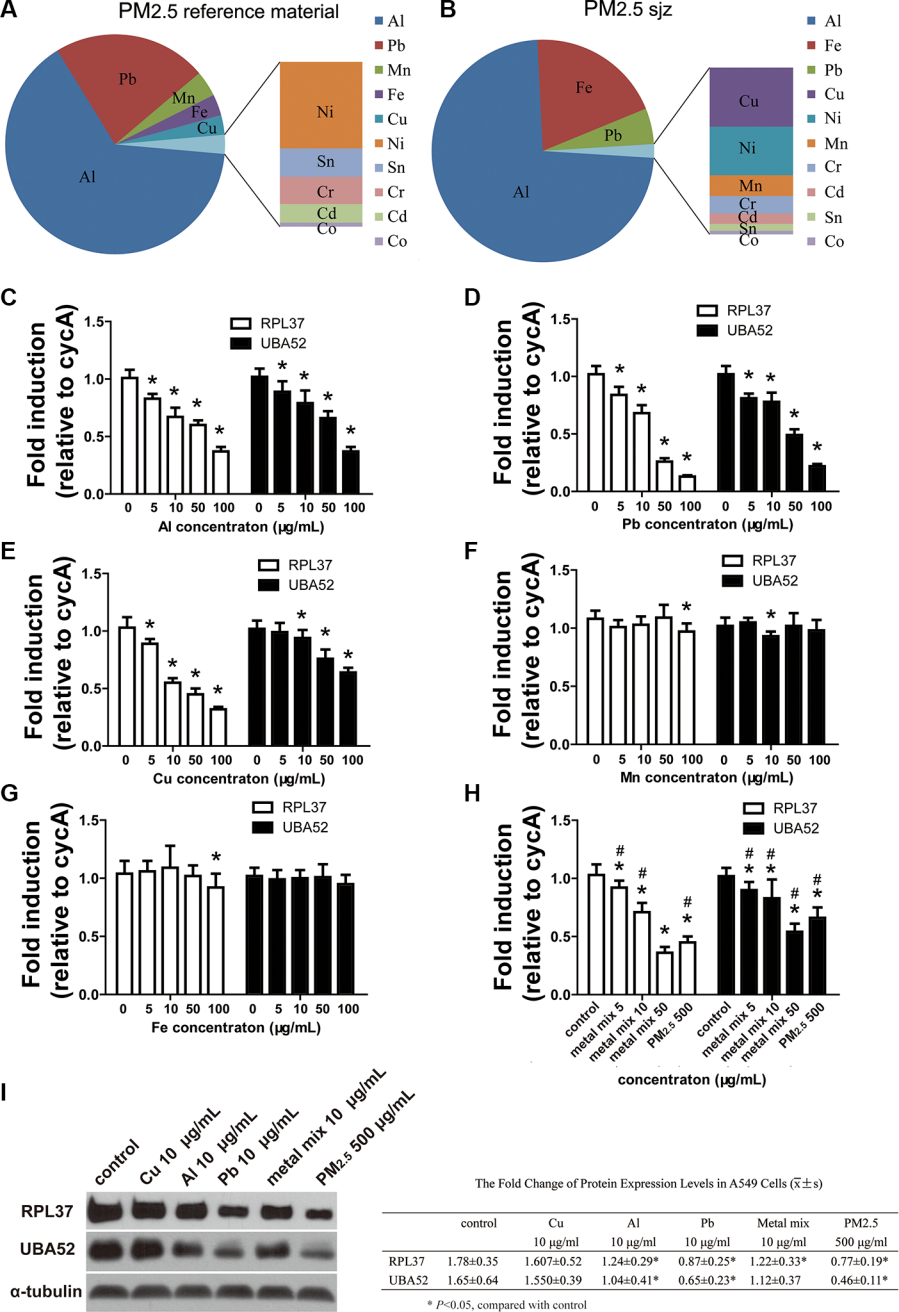
Expression of miR-4516 target genes as inhibited by PM_2.5_ and PM metals Proportion of different metal components in PM_2.5_ reference material. (**B**) Proportion of different metal components in PM_2.5sjz_. mRNA expression levels of RPL37 and UBA52 were modulated by different doses of (**C**) Al, (**D**) Pb, and (**E**) Cu metal particles. mRNA expression levels of RPL37 and UBA52 were slightly modulated by (**F**) Fe or (**G**) Mn metal particles. (**H**) mRNA expression levels of RPL37 and UBA52 were inhibited by the Al, Pb, and Cu metal mix and PM_2.5_. (**I**) Immunoblot analysis demonstrated the down-regulation of RPL37 and UBA52 protein expression induced by PM_2.5_, as well as the Al and Pb metal particles. *P < 0.05, compared with control, ^#^*P* < 0.05, compared with 500 μg/mL PM_2.5_ treatment group.

ICP-MS was used to analyze the mass concentration of Al, Pb, Mn, Fe, Cu, Ni, Sn, Cr, Cd, and Co in PM_2.5_ reference material, and the corresponding concentrations (w/w) were 12.71 ± 0.58, 4.43 ± 0.46, 0.74 ± 0.09, 0.61 ± 0.11, 0.56 ± 0.08, 0.29 ± 0.08, 0.09 ± 0.02, 0.09 ± 0.02, 0.06 ± 0.01, and 0.02 ± 0.01 μg/mg, respectively. The proportion of different metal components is shown in Figure 3A. The mass concentrations of SO4^2−^, NO_3_^−^, NH_4_^+^, OC, and EC in PM_2.5sjz_ were 46.5 ± 12.7, 24.1 ± 10, 21.3 ± 9.6, 136.6 ± 31.9, and 21.4 ± 7.2 μg/m^3^. The mass concentrations (w/w) of Al, Fe, Pb, Cu, Ni, Mn, Cr, Cd, Sn, and Co in PM_2.5sjz_ were 17.81 ± 2.06, 4.77 ± 0.67, 1.27 ± 0.37, 0.17 ± 0.05, 0.14 ± 0.04, 0.06 ± 0.01, 0.05 ± 0.01, 0.03 ± 0.02, 0.03 ± 0.01, and 0.01 ± 0.00 μg/mg. Notably, Al, Fe, and Pb contributed to the most weight proportion of PM_2.5sjz_ (Figure 3B). Therefore, the major metal components of PM_2.5_ reference material could partially mimic PM_2.5sjz_.

We further assessed the effects of PM_2.5_, Al, Pb, Cu, Fe and Mn particles on the expression levels of miR-4516 targets RPL37 and UBA52. As shown in Figures 3C–3E, the expression levels of RPL37 and UBA52 mRNA were down-modulated in a dose-dependent manner by exposure to 5, 10, 50, or 100 μg/ml of Al, Pb, and Cu particles, respectively. Since the trends of modulation induced by Mn and Fe were inconsistent with other metal particles (Figure 3F and 3G). The Al, Pb, and Cu particles demonstrated notable down-modulation of RPL37 and UBA52; we mixed these three particles according to their ratio of weight in PM_2.5_ reference material. As shown in Figure 3H, the 50 μg/ml metal mix demonstrated enhanced inhibitory effects on UBA52 expression and similar inhibition of RPL37 in A549 cells, when compared with 500 μg/ml PM_2.5_ treatment. Expression of RPL37 and UBA52 in A549 cells exposed to 5 or 10 μg/mL of the metal mix were significantly lower than controls. However, 500 μg/mLl PM_2.5_ demonstrated notable inhibition on expression of RPL37 and UBA52 than 5 or 10 μg/mL metal mix (Figure 3H). Protein expression levels of RPL37 and UBA52 were confirmed by immunoblots. As shown in Figure 3I, blunting expression of RPL37 and UBA52 were observed after treatment with 10 μg/ml Al, 10 μg/ml Pb, 10 μg/ml metal mix, or 500 μg/ml PM_2.5_ for 24 h.

### Autophagy is implicated in PM_2.5_-induced cellular damage

**Figure 4:**
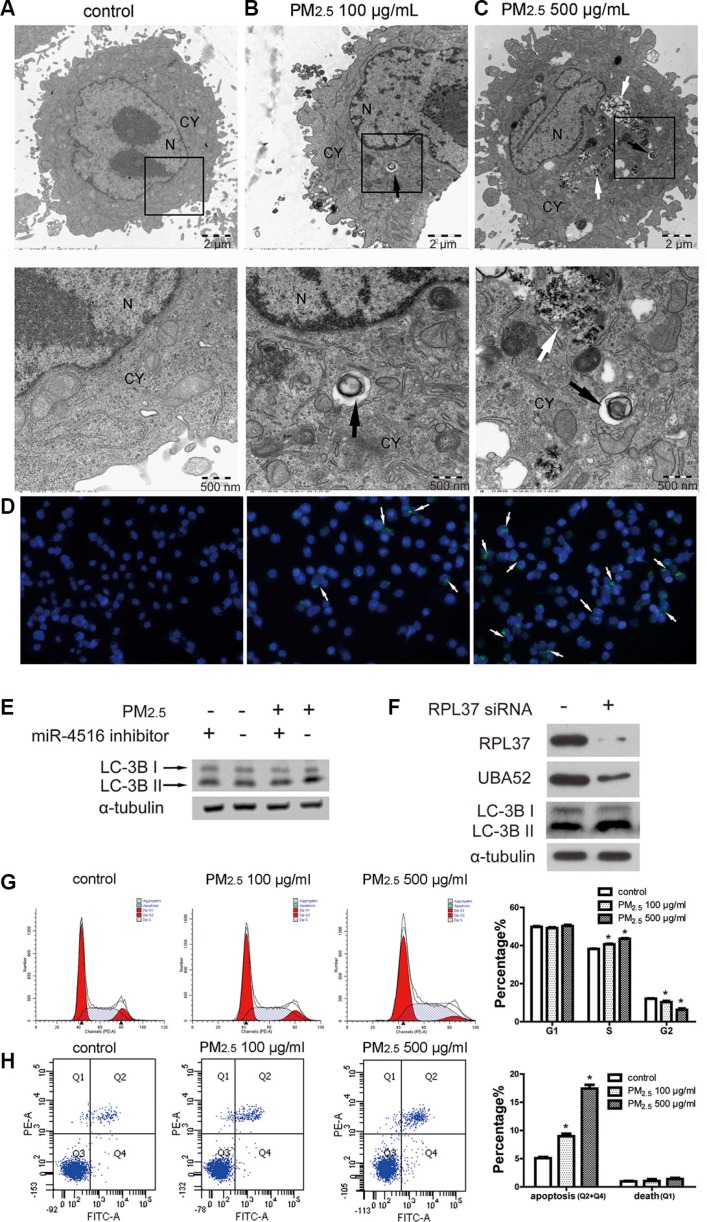
PM_2.5_ treatment induced autophagy in A549 cells as regulated by miR-4516 and its target gene (**A**) A549 cells were treated with control medium for 24 h, no autophagosomes were observed in the cytoplasm. A549 cells were treated with (**B**) 100 μg/mL or (**C**) 500 μg/mL PM_2.5_ for 24 h. Typical autophagosomes with double membranes and cellular contents were observed in the cytoplasm, as shown by the black arrow. N, nucleus, CY, cytoplasm. Insets are magnified below each image highlighted with black box). Black arrows show typical autophagosome and black arrows show intracellular PM. (**D**) Increased number of autophagic vacuoles were observed in A549 cells. Autophagic vacuoles were showed by white arrows. (**E**) Expression levels of LC3B-I and LC3B-II were increased in A549 cells after treatment with 100 μg/ml PM_2.5_ for 24 h. Co-treatment of PM_2.5_ and the miR-4516 inhibitor could reverse the increased expression of LC3B-I and LC3B-II. (**F**) Expression levels of RPL37 and UBA52 were inhibited after RPL37 siRNA transfection in A549 cells. Meanwhile, increased expression of LC-3B-I and -II were also observed. (**G**) S stage arrest was induced by PM_2.5_ exposure for 24 h. (**H**) The proportion of apoptosis in A549 cells was significantly increased after PM_2.5_ treatment for 24 h.

Autophagy has been associated with PM_2.5_-induced pulmonary dysfunction, including human bronchial epithelial (HBE) cells [28] and human A549 cells [29, 30]. We further investigated whether miR- 4516 and its target gene were involved in autophagy induced by PM_2.5_. Figure 4A shows the normal structure of control-treated A549 cells. Autolysosomes containing cellular debris, which were formed through the fusion of lysosome with the outer membrane of an autophagosome, were observed in cytoplasm of A549 cells exposed to 100 or 500 μg/mL PM_2.5_ for 24 h (Figures 4B and 4C), as shown by black arrows. Meanwhile, in the high-dose PM_2.5_-treated A549 cells, the uptake of massive particles could be observed in the cytoplasm (Figure 4C) as shown by white arrows. The autophagic flux in A549 cells was evaluated by fluorescent dye, which could be incorporated into autophagosomes and autolysosomes. Enhanced green fluorescence demonstrated an increase in the number of autophagic vacuoles in A549 cells following PM_2.5_ treatment (Figure 4D). As shown in Figure 4E, the expression levels of LC3B-I and LC3B-II were augmented after PM_2.5_ treatment. Furthermore, co-treatment of PM_2.5_ and the miR-4516 inhibitor limited LC-3B-II expression. The inhibited UBA52 expression and the enhanced LC3B-I and LC3B-II expression were induced by RPL37 blunting (Figure 4F). Figure 4G suggests arrests in the S phage of A549 cells, and the proportion of apoptosis cells was enhanced (Figure 4H) after PM_2.5_ exposure.

### Expression levels of miR-4516 in serum are positively associated with the metal burden

**Figure 5:**
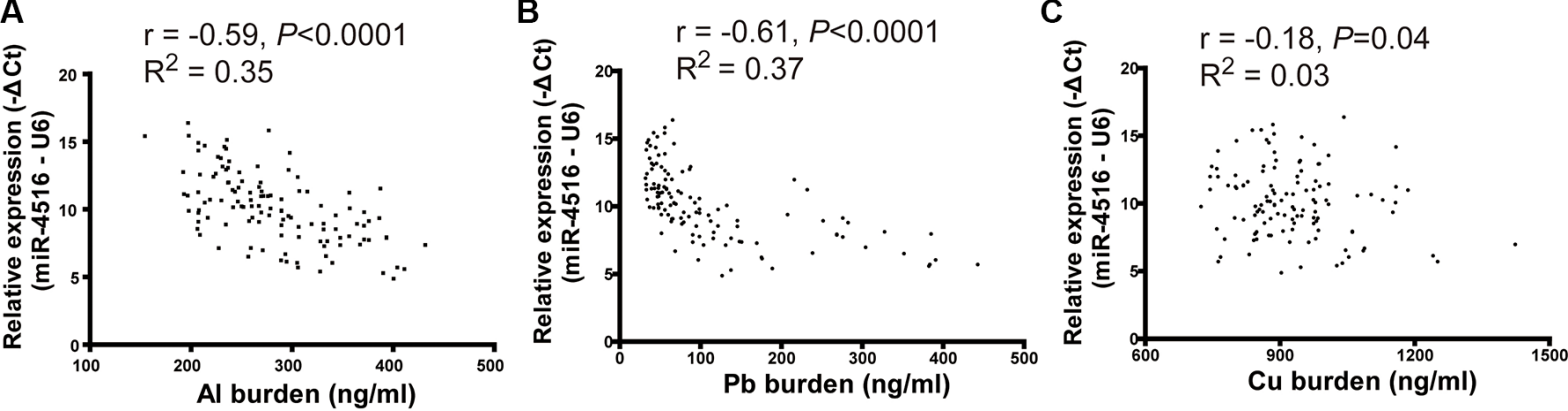
Scatter plot of miR-4516 and metal levels in serum (**A**) Expression of miR-4516 was negatively associated with the Al burden in serum of individuals from a moderately polluted city. (**B**) Expression of miR-4516 was negatively relative to the Pb burden in serum of individuals from a moderately polluted city. (**C**) Expression of miR-4516 was slightly affected by Cu levels in serum samples.

The relative expression of miR-4516 and different metal concentrations were determined in serum samples of individuals living in a city with moderately polluted air to explore the actual association between miR-4516 and the internal metal burden. miR-4516 expression was significantly correlated with Al burden (*R* = −0.59,*P* < 0.01), Pb burden (*R* = −0.61, *P* < 0.01), and Cu burden (*R* = −0.18, *P* = 0.04; Figure 5).

**Figure 6:**
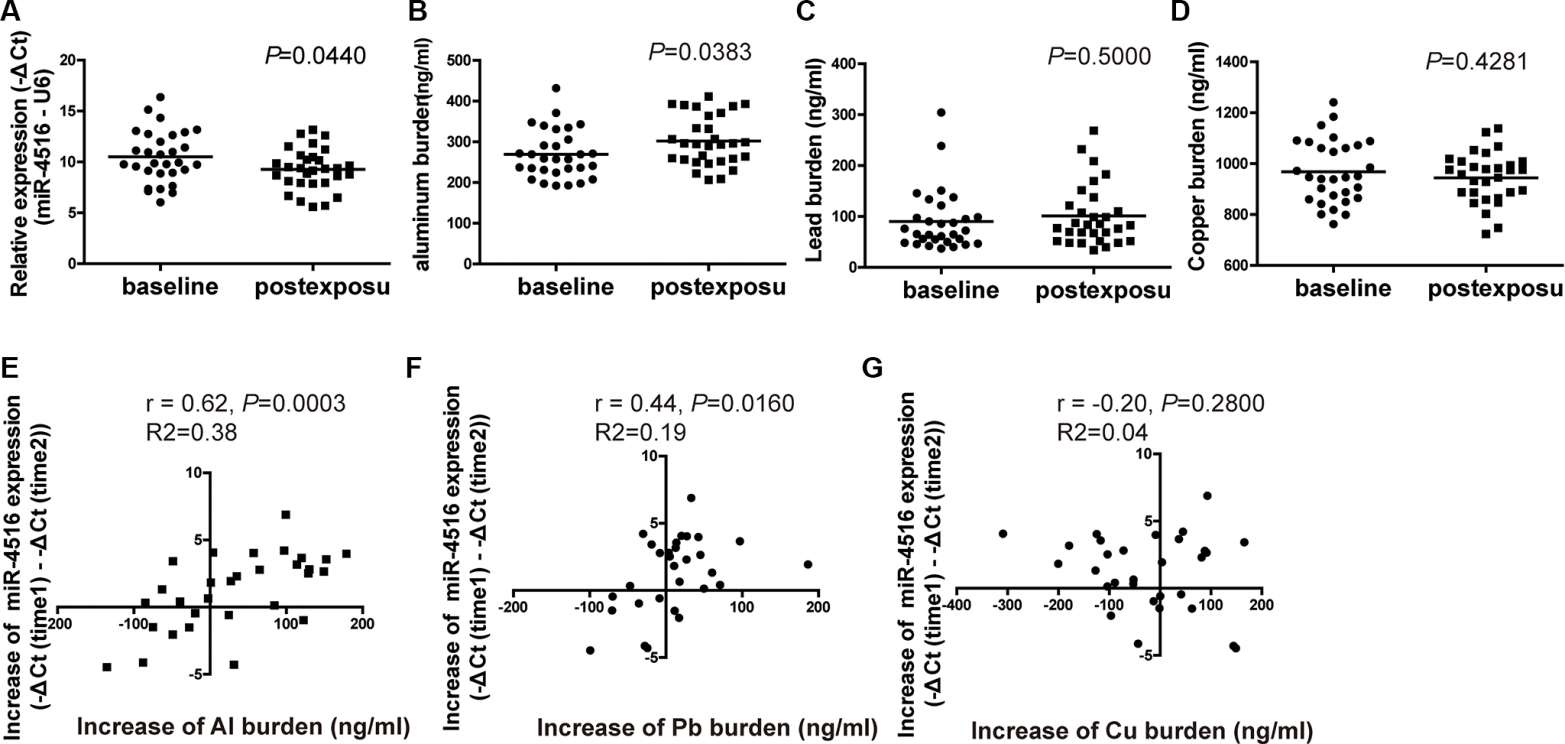
Alterations of miR-4516 expression and the metal burden between baseline and post-exposure levels (**A**) Relative expression level of miR-4516 and (**B**) Al burden were significantly increased after 3 months in individual sera. *P < 0.05, compared with baseline level. (**C**) Pb and (**D**) Cu burdens were not significantly modulated after 3 months. Increment of miR-4516 expression was positively associated with the increment of (**E**) the Al burden and (**F**) Pb burden. (**G**) No association between the increment of miR-4516 and that of the Cu burden was found.

Winter is the season with the most air pollution compared with other seasons in Shijiazhuang City because of climate conditions and thermal energy requirements. Therefore, the alteration of miR-4516 expression levels and the internal metal burden of 30 individuals were detected between the baseline and post exposure levels. After 3 months, the expression levels of miR-4516 and Al burden significantly increased (Figures 6A and 6B). No differences were found in the Pb and Cu burdens compared with the baseline with post-exposure levels (Figures 6C and 6D).

The levels of miR-4516 and metal burden were modulated after 3 months of PM_2.5_ exposure; thus, the correlation between the increments of miRNA and metal concentrations was evaluated by Pearson correlations analysis. The increase in miR-4516 expression level between baseline and post-exposure was positively correlated with increased levels of Al burden (*R* = 0.62, *P* = 0.0003; Figure 6E) and Pb burden (*R* = 0.44, *P* = 0.016; Figure 6F) between initial and second assessment, but were not associated with Cu burden (*R* = −0.20, *P* = 0.28; Figure 6G). The increased circulating miR-4516 level was not relative to individual characteristics, such as sex and age (Table S6).

## DISCUSSION

Based on bioinformatics analysis coupled with cellular assays, the present study demonstrated that the miR-4516/RPL37-dependent metal binding pathway plays a role in responses to PM_2.5_ metal exposure. PM_2.5_ exposure could induce autophagy in a pulmonary cell model of A549 cells, which is mediated by ribosomal reduction. In addition, miR-4516 expression levels were significantly positively correlated with individual Al and Pb burdens *in vivo*.

Although accumulative studies focused on miRNA expression in response to environmental stimuli, including PM_2.5_ or other air pollutants, few studies evaluated changes in miRNA involved in PM metal components [31–32]. Black carbon and organic carbon of PM were negatively associated with microRNA levels of elderly men [31]. An *in vitro* study suggested that the regulation of miR-182 and miR-185 to its target genes SLC30A1, SERPINB2, and AKR1C1, were induced by PM_2.5_ exposure. Furthermore, increased expression of these three target genes were detected in human lung cancer [32]. A recent study showed that miR-222 and miR-21 were significantly increased following exposure to metal-rich particulate matter [18]. Briefly, miR-222 and miR-21 were investigated according to their potential regulations upon inflammation and oxidative stress. However, the underlying mechanism of candidate miRNAs responding to PM or metal components was not elucidated.

We proposed that miR-4516 as a candidate miRNA is involved in PM metal component exposure. Public databases, such as mirWalk or Targetscan, are available to identify potential miRNA target sites in 3′-untranslated region (3′-UTR) of a protein encoding gene. However, potential targets of miRNAs generally include thousands of genes [18]. Therefore, functional analysis of miRNAs based on predicted target genes alone is not sufficient. In terms of accuracy, proteomic analysis was used to confirm the modulation of miR-4516 targets in the present study. By applying the bioinformatics strategies, seven proteins were associated with the metal binding pathway; among which, RPL37 and UBA52 had protein–protein interactions and involved the ribosome pathway.

Ribosomes are essential organelles of protein synthesis, and ribosomal proteins are associated with extra-ribosomal functions, such as cell proliferation, differentiation, apoptosis, and other processes [33]. The down-regulation of ribosomes at the protein level is a stress response to environmental stimuli [34, 35]. In the present study, bioinformatics analysis coupled with cell-based assays implied the connection between the metal ion binding pathway and the ribosome pathway because the miR-4516 target genes, namely, RPL37 and UBA52, are involved in both pathways. The down-regulation of ribosomal proteins in A549 cells could be a cellular response to PM or metal components stresses.

The disturbance of ribosomal proteins has been linked to pathological processes. RPL37 is a component of the 60S subunit of ribosomes and belongs to the L37E family of ribosomal proteins. UBA52, also named RPL40, encodes the ubiquitin A-52 residue ribosomal protein fusion product 1. Down-regulation or depletion of RPL37 is a well-established inducer of ribosomal stress, which has been shown to decrease MdmX protein levels and activate p53 [36–38]. Ribosomal stress induced autophagy has been reported in Arabidopsis [39] and yeast [34]. In eukaryotic cells, autophagy probably contributes to the proteome turnover while protecting the degradation of ribosomes [40]. In the present study, we observed the simultaneous occurrence of autophagy and disturbance of ribosomes. Furthermore, the depletion of RPL37 by siRNA in A549 cells enhanced the expression of LC3B, a critical hallmark of autophagy in mammal cells.

Autophagy is critical to maintain cellular homeostasis, as well as constitutive protein and organelle turnover [41]. Cellular stress induced by xenobiotic exposure could cause autophagy as a cytoprotective mechanism. However, the dysfunction of autophagy contributes to diverse human diseases [41]. Autophagy is reported to play a critical role in pulmonary toxicity induced by environmental toxicants, such as increased air pollution [28–30]. In these studies, autophagy was confirmed to be activated in cell death or pulmonary damages. Notably, autophagy and lysosomal degradation were required for PM-induced inflammatory responses via the activation of NFKB1 and AP-1 pathways in HBE cells. Therefore, autophagy inhibition is suggested for the treatment of airway disorders induced by airborne PM [28]. Deng et al. [29] suggested that dysfunction of autophagy could trigger loss of cell viability in A549 cells exposed to different doses of PM_2.5_. Our results showed that PM_2.5_ exposure-induced autophagy in A549 was regulated by miR-4516 and could be a result of ribosome reduction. This miR-4516/RPL37 pathway-related autophagy is involved in the cellular response to PM_2.5_ and PM metal components.

Our subsequent study based on population living in a city with moderate air pollution confirmed the association between miR-4516 and PM or PM metal exposure. Bollati et al. [18] reported that expression of miR-222 and miR-21 was significantly increased in an occupational population exposed to Pb- or Cd-rich PM; this association was limited to non-smokers. We investigated 30 non-smoking subjects to study the alteration of miR-4516 expression after winter season PM exposure. Results showed that increased expression level of miR-4516 is positively regulated by enhanced Al and Pb burden *in vivo*. The identification of individuals who have enhanced responses to PM may suggest potential mechanisms of physiological dysfunction and provide candidate markers that can be used for more detailed risk assessment.

One limitation of our study is that individual PM_2.5_ or the PM metal components exposure could not be well-characterized. The increase in metal burden could also be attributed to occupational or indoor exposure. The limited number of study subjects and duration of observation might affect the association between the metal burden and miRNA expression. The other limitation is that only one cell model was used in the present study. Further validation of biological effects induced by PM metal components by other pulmonary cell models is required.

In summary, out findings suggest that PM exposure, particularly the metal components of Al and Pb, can modulate miR-4516 expression. The possible underlying mechanisms include ribosome reduction-mediated autophagy. Further studies are required to determine the effects of such alterations on human health.

## MATERIALS AND METHODS

### Study subject

We recruited 120 non-smoking, healthy individuals, including 60 males (mean age, 46 years; range 30–61 years) and 60 female (mean age, 44 years, range 30–59 years) in a tourist city, Zhangjiakou, Northern China and 120 healthy individuals, including 60 males (mean age, 46 years; range 30–60 years) and 60 females (mean age, 42 years; range 30–60 years) in an industrial city, Shijiazhuang, Northern China. These individuals were free of cancer, cardiovascular disease, and pulmonary disease; all of them had been living in their current city for at least a year. We obtained serum and whole blood samples for miRNA and metal burden analysis from November 2014 to February 2015, with informed consent and agreement.

The other 30 healthy individuals (15 male, mean age, 44 years, range 31–59 years; 15 female, mean age, 45 years, range 33–60) were recruited from moderately air polluted city of Shijiazhuang. These individuals were free of cancer, cardiovascular disease, and pulmonary disease, and all had been living in their current city for at least 1 year. We excluded those who were current active or passive smokers (living with a current smoker) and former smokers (quit smoking for at least 3 years). We obtained serum and whole blood samples for miRNAs and metal burden analysis at two different times: initially, baseline samples collected from 1st to 5th of November, 2014; followed by post-exposure samples collected from 1st to 5th of February 2015, with informed consent and agreement.

The research protocol was approved by the ethics review board of Southeast University (NO. 2014070012) and all of the samples were used in compliance with corresponding ethical regulations. Serum was separated from the half volume of blood within 1 h by centrifugation at 3000 × g for 10 min, followed by 15 min high-speed centrifugation at 12000 × g to completely remove the cellular debris. The supernatant serum was collected, as well as the other half volume of whole blood, and stored at −80°C until subsequent use.

According to emission inventory data from the Bureau of Meteorology of China, the mean air pollution index (API) and PM_2.5_ levels during winter (from December 2014 to February 2015) in Zhangjiakou City were 56.21 and 29.51 μg/m^3^, respectively. The mean API and PM_2.5_ levels during autumn (from September to November 2014, baseline level) in Shijiazhuang City were 129.97 and 96.01 μg/m^3.^ The API and PM_2.5_ levels during winter (from December 2014 to February 2015; post-exposure level) in Shijiazhuang City were 213.44 and 122.51 μg/m^3^, respectively. The air pollution levels in Zhangjiakou (winter) and Shijiazhuang (baseline or post-exposure level) were in compliance with the API standards of good, slight pollution, or moderate pollution, respectively.

### Fine particulate matter and metal particles

Urban particulate matter (PM_2.5)_ (SRM 1648a) was purchased from the National Institute of Standards and Technology (NIST; USA). This standard reference material (SRM) is atmospheric particulate matter collected in an urban area. All constituents provided in SRM 1648a were naturally present in the material before processing. The major components of SRM 1648a were introduced by a research group from NIST [42].

Fine-particle samples of Shijiazhuang city (PM_2.5sjz_) were collected with a TH-1000C II air sampler (Tianhong, Wuhan, China) with Teflon-coated glass fiber filters at a flow rate of 1.00 m^3^/min from ambient air of a downtown area in Shijiazhuang, China. The samples were collected for 7 days (from the 12th to 17th) of each months (from December 2014 to February 2015) and at 24 h for each day. Each filter was cut into small pieces, added with 15 ml of pure water and treated with ultra-sonication three times for 15 min at 4°C. The PM_2.5sjz_ samples were dried by lyophilization and stored at 4°C.

The water soluble inorganic ions (SO_4_^2−^, NO_3_^−^, and NH_4_^+^), EC/OC, and metal elements of 21 samples were detected by ion chromatography (ICS-90, Dionex, USA), optical carbon analysis (DRI model 2001A, USA) and inductively coupled plasma mass spectrometry (ICP-MS, Agilent 7700, USA), respectively.

Fine metal particles of Al, Pb, Mn, Fe, and Cu were purchased from Shanghai Naiou Nano Technology Co., Ltd, China. The size distributions of metal particles were evaluated by a zetasizer (nano-zs90, Malvern Instruments, UK). The mean diameters of metal particles were 632.7 nm (Cu particles), 964.2 nm (Al particles), 1242 nm (Pb particles), 511.7 nm (Mn particles), and 683.5 nm (Fe particles), as shown in Figure S1. All the aerodynamic diameters of metal particles were less than 2.5 μm.

### miRNA microarray analysis

The human lung adenocarcinoma cell line A549 (American Type Culture Collection) was maintained in Dulbecco's modified Eagle's medium (DMEM) at 37°C in 5% CO_2_. Cells were seeded in 10 cm culture dishes and exposed to 500 μg/mL PM_2.5_ with three biological replicates. Control groups were treated with culture medium. Total RNA was extracted with the TRIZOL reagent (Invitrogen, 15596-026, USA) according to the manufacturer's instructions.

The microarray analysis for miRNA profiling was conducted by the miRCURY LNA Array system (Exiqon, Vedbaek, Denmark). Raw data were subjected to background subtraction and normalization with the *limma* R-package [43]. Discriminant miRNAs and differences between groups were analyzed using Bayes moderated *t*-test (limma) with Benjamini Hochberg false discovery rate (FDR) at *P* < 0.05, unless otherwise specified. A two fold cut-off was applied to select up-regulated and down-regulated miRNAs.

### Proteomics analysis

A549 cells were cultured as previously described, lysed in RIPA buffer containing a protease inhibitor cocktail (Cell Biolabs, AKR190, USA). Extracted proteins were subsequently tagged with tandem mass tags for quantitative mass spectrometry (TMT^®^ Mass Tagging Kit, 90063, Thermo Scientific, Germany). Raw data of liquid chromatography–mass spectrometry (LC-MS/MS) were assessed with Sequest-HT as a search engine within the Proteome Discoverer version 1.4 against the Human RefSeq database (71465 proteins, updated on 03/03/2014).

### Functional group analysis

Significantly modulated miRNAs were ranked according to the *P* value. The possible binding sites of the top 1 modulated miRNA were predicted by the online database miRWalk [27]. The predicted mRNA, whose encoding proteins had a FC greater than 1.5 according to proteomics analysis, were further analyzed using a functional annotation tool Database for Annotation, Visualization, and Integrated Discovery (DAVID 6.7). All known PPIs from BioGRID, DIP, HPRD, and String were combined to explore the potential interactions among target proteins.

### Confirmation of miRNA expression levels in A549 cells and individual serum

A549 cells were seeded in 10 cm culture dishes treated with 50, 100, 250, or 500 μg/mL PM_2.5_ for 24 h. Total RNA was isolated according to the instructions provided by the manufacturer of TRIZOL. The total RNA from human serum was extracted using miRNeasy Serum/Plasma Kit (Qiagen, 217184, Germany). The expression levels of miR-4516 were amplified with PCR primers (RiboBio, China) on a Quant Studio 6 Flex system (Applied Biosystems, Life Technologies, USA). The relative expression levels of miRNAs were normalized against U6 and were calculated using the 2^−ΔΔCt^ method. All of the experiments were performed in triplicates.

### 3′-UTR luciferase reporter assay

The wild type and mutant 3′-UTRs of RPL37 were cloned into the pmiR-RB miRNA reporter vector (Ribobio, GUR100008-P-2, China) to validate the regulation between miR-4516 and RPL37. The wild type contained binding sites of RPL37 3′-UTR with miR-4516. The complementary sequence to the binding sites was replaced by GAAGAGG for mutagenesis. Sequence of *Renilla* luciferase (Rluc) and firefly luciferase (Luc) were constructed in plasmid vectors for reporter fluorescence (Rluc) and internal reference (Luc), respectively. A549 cells (1 × 10^4^), vectors (100 ng), and the miR-4516 mimic (1.5 pmol) were mixed in a 96-well plate and cultured for 48 h. Luciferase activity was measured by a dual-luciferase reporter assay system (Promega, TM040, USA) and determined by Mithras LB 940 (Berthold Technologies, Belgium). The luciferase signal ratio (Rluc/Luc) was calculated.

### Validation of miR-4516 target gene expression in A549 cells

A549 cells were seeded in 10 cm culture dishes and exposed to 5, 10, 50, or 100 μg/mL Al, Pb, Cu, Mn, Fe, or control medium for 24 h. Additional metal mixtures of Al, Pb and Cu were prepared with ratio as Al:Pb:Cu = 24:9:1. A549 cells were seeded in 10 cm culture dishes and exposed to 500 μg/mL PM_2.5,_ 5, 10, or 50 μg/mL metal mixture or control medium for 24 h. Cells were harvested for total RNA and protein extraction. qRT-PCR assays were performed as description [44]. The primer sequences are: RPL37 forward 5′-TCGCAATAAGACGCACACGTT-3′ and 5′-CTCATTCGACCAGTTCCGGT-3′, UBA52 forward 5′-AAGACAAGGAGGGTATCCCAC-3′, UBA52 reverse 5′-TGTTGTAGTCTGAGAGAGTGCG, and cyclophilin A forward 5′-CCCACCGTGTTCTTCGACATT-3′, reverse 5′-GGACCCGTATGCTTTAGGATGA-3′. All of the experiments were performed in triplicate. The mRNA levels were relative to cyclophilin A for the indicated gene.

### miR-4516 inhibitor transfection and siRNA knockdown in A549 cells

A549 cells were transfected with Lipofectamine RNAiMAX (Life Technologies, 13778150, USA) in 10 cm culture dishes with a final concentration of 100 nM miR-4516 inhibitor or miR-NC (RiboBio Corporation, China). At 48 h post-transfection, cells were treated with 100 μg/ml PM_2.5_ for another 24 h and then harvested for total RNA and protein extraction.

For siRNA knockdown, we transfected A549 cells in a 6-well plate with siRNAs (Thermo Fisher Scientific, USA) directed against control or RPL37 with DharmaFECT1 (Thermo Fisher Scientific Dharmacon, T-2001, USA). At 48 h post-transfection, cells were harvested for Western blotting analysis.

### Protein extraction and Western blot analysis

A549 cells were seeded in 10 cm culture plates and treated with 0, 100, and 500 μg/ml PM_2.5_, as well as 50 μg/ml Al, Pb, or Cu of fine particles for 24 h then lysed by RIPA lysis buffer (1 ml for 10^7^ cells) and the concentration of total proteins was determined by Coomassie-Plus protein assay reagent (Pierce, 23238, USA). Untreated A549 cell protein extracts were used for co-immunoprecipitation (CO-IP) assays, where proteins were incubated with primary antibodies of RPL37 (1:1000 dilution; Abcam, ab207562, USA), UBA52 (1:1000 dilution, Abcam, ab109227, USA), and IgG (1:10000, Abcam, ab109489, USA). Proteins of A549 cells with different treatments were analyzed by immunoblots with primary antibodies for the following antigens: human LC3B (1:1000 dilution; CST, 2775, USA), RPL37 (1:1000 dilution; Abcam, USA) and a-tubulin (1:10 000 dilution; Sigma, USA). A semi-quantitative analysis of Western blot was performed by Image Lab 3.0 (BIO-RAD, USA). The grayscale of each blot was measured five times and normalized to a-tubulin.

### Endogenous protein immunoprecipitation

Immunoprecipitation of endogenous proteins was accomplished with a Universal CO-IP kit (Active Motif, 54002, USA). A549 whole cell extracts were first incubated with protein A agarose beads. Cleared supernatants were incubated with RPL37, UBA52, or normal human IgG for 2 h before addition of protein A to the agarose beads. After binding, beads were pelleted by centrifugation and washed with the buffer. After washing, immunoprecipitated materials were eluted and immunoblotted with anti-human UBA52 (1:1000 dilution) or RPL37(1:1000 dilution) primary antibodies.

### Transmission electron microscopic observation

A549 cells were treated with 100 or 500 μg/ml PM_2.5_ for 24 h, collected, and fixed with 2.5% glutaraldehyde in 0.1 M sodium dihydrogen phosphate (pH 7.4). The samples were fixed in 1% OsO_4_ for 1 h, stained with uranyl acetate and lead citrate, and observed under a transmission electron microscope (JEOL-1010, Japan).

### Alteration of cellular phenotypes

The Cyto-ID autophagy detection kit (Enzo, USA) was used to determine the presence of autophagic vacuoles and monitor autophagic flux in A549 cells according to the manufacturer's instructions. Cells were exposed to 0, 100, or 500 μg/mL PM_2.5_ for 24 h and collected for centrifugation at 400 × *g* for 5 min and washed once by PBS. The pellet was resuspended in 500 μL of the CytoID green detection reagent, incubated for 30 min in the dark at 37°C, and subsequently analyzed by a fluorescent microscope.

Cell cycle and apoptosis were detected with a FACS Calibur Flow Cytometry apparatus (BD Biosciences, USA) [45]. A549 cells were fixed in 75% ethanol and stained with propidium iodide (PI) for cell cycle analysis. Annexin V– FITC staining was used to detect occurrence of apoptosis.

### Data analysis

Data were expressed as mean ± standard error of the mean (SEM). Statistically significant differences were determined by one-way ANOVA followed by Dunnett's multiple comparison tests for the luciferase reporter assay. The miR-4516 expression between different age levels, the miR-4516, RPL37, and UBA52 mRNA expression increased in cellular assays. The 2^−ΔΔCt^ method was used to analyze the qRT-PCR results in cellular experiments. The −ΔCt method was used to express the results of qRT-PCR in individual serum samples. Student's *t*-test was performed to determine the differences of miR-4516 expression between two cities, between male and female individuals, between time 1 and time 2, and the different Al, Pb, and Cu burden between time 1 and time 2. The relationship between miR-4516 and the metal burden was analyzed by the Pearson correlation coefficient. Statistical analysis was performed with SPSS 12.0 and the significance was set at *P* < 0.05.

## SUPPLEMENTARY MATERIALS FIGURES AND TABLES


