# Supplementary material for: Role of microRNA-4516 involved autophagy associated with exposure to fine particulate matter
Source: Oncotarget. 2016 Jun 13;7(29):45385–97. doi: 10.18632/oncotarget.9978 (PMC5216729; doi:10.18632/oncotarget.9978)
Supplement: Supplementary file 1 [file oncotarget-07-45385-s001.pdf]

Role of microRNA-4516 involved autophagy associated with exposure to fine particulate matter

Supplementary Materials

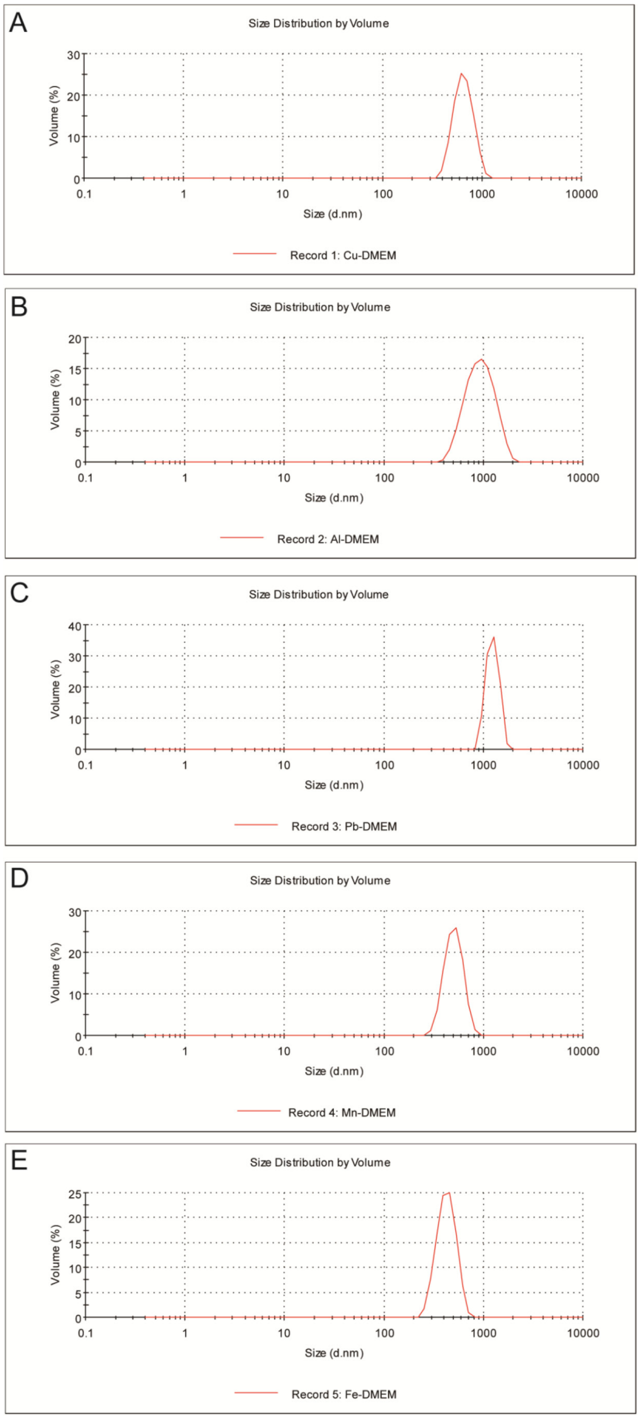

Supplementary Figure S1: Size distribution of metal particles. The metal particles were suspended in DMED culture medium with a final concentration of 1 mg/ml, and dispersed by ultrasonication for 15 min. Then the size distribution of metal particles were immediately evaluated by a zetasizer (nano-zs90, Malvern Instruments, UK). (**A**) Mean size of Cu particles is 632.7 nm. (**B**) Mean size of Al particles is 964 nm. (**C**) Mean size of Pb particles is 1242 nm. (**D**) Mean size of Mn particles is 511.7 nm. (**E**) Mean size of Fe particles is 683.5 nm.

Supplementary Table S1: miRNA microarray predicted modulation of miRNA in $PM_{2.5}$ treated A549 cells

microRNA	Fold change	FDR
hsa-miR-4516	9.687	0.031
hsa-miR-4732-5p	5.867	0.034
hsa-miR-4778-5p	5.563	0.037
hsa-miR-4449	5.352	0.014
hsa-miR-3201	5.278	0.014
hsa-miR-675-5p	4.360	0.037
hsa-miR-638	3.944	0.014
hsa-miR-1469	3.907	0.014
hsa-miR-204-3p	3.212	0.037
hsa-miR-4750-5p	2.967	0.037
hsa-miR-3940-5p	2.848	0.014
hsa-miR-652-5p	2.710	0.041
hsa-miR-3611	2.660	0.022
hsa-miR-802	0.172	0.049
hsa-miR-4319	0.246	0.037
hsa-miR-1322	0.253	0.049
hsa-miR-3176	0.260	0.014
hsa-miR-933	0.260	0.037
hsa-miR-4536-3p	0.317	0.034
hsa-miR-4445-3p	0.345	0.049

Supplementary Table S2: miR-4516 expression by individuals' characteristics

		good air quality city			Moderate air pollution city	
**Variable**	***n***	**miR-4516 (mean, 95% CI)**	***P*-value**	***n***	**miR-4516 (mean, 95% CI)**	***P*-value**
Age, years						
< 40	38	13.87 (13.29, 14.45)		42	9.776 (8.941, 10.61)	
40–50	44	13.38 (12.87, 13.89)		40	9.771 (8.978, 10.56)	
> 50	38	13.32 (12.75, 13.90)	0.3144	38	10.86 (10.03, 11.70)	0.1022
Sex						
Male	60	13.48 (13.01, 13.96)		60	9.713 (9.075, 10.35)	
Female	60	13.50 (13.07, 13.93)	0.9590	60	10.52 (9.830, 11.21)	0.0881

Supplementary Table S3: Down-regulations of proteins encoded by miR-4516 target genes

Gene symbol	mRNA encoded protein function	Fold Change: Low dose $PM_{2.5}$	Fold Change: High dose $PM_{2.5}$
RPL37	60S ribosomal protein L37	0.865	0.39
FSTL1	follistatin-related protein 1 precursor	0.895	0.666
PER1	PREDICTED: period circadian protein homolog 1 isoform X2	0.75	0.593
U2AF2	splicing factor U2AF 65 kDa subunit isoform b	0.521	0.33
AKAP17A	A-kinase anchor protein 17A isoform 1	0.82	0.633
PRKCB	protein kinase C beta type isoform 1	0.958	0.614
SUB1	activated RNA polymerase II transcriptional coactivator p15	0.734	0.449
BET1	BET1 homolog	0.729	0.595
LIX1L	LIX1-like protein	0.925	0.645
CSF1	macrophage colony-stimulating factor 1 isoform c precursor	0.769	0.576
PPP2R3A	serine/threonine-protein phosphatase 2A regulatory subunit B" subunit alpha isoform 3	0.854	0.335
PRPF38B	pre-mRNA-splicing factor 38B	0.652	0.598
LUC7L	putative RNA-binding protein Luc7-like 1 isoform b	0.464	0.213
POLR1D	DNA-directed RNA polymerases I and III subunit RPAC2 isoform 3	0.954	0.557
PLOD2	procollagen-lysine,2-oxoglutarate 5-dioxygenase 2 isoform 1 precursor	0.75	0.659
MTUS1	PREDICTED: microtubule-associated tumor suppressor 1 isoform X3	0.734	0.655
UBA52	ubiquitin-60S ribosomal protein L40 precursor	0.858	0.31
RSRC2	PREDICTED: arginine/serine-rich coiled-coil protein 2 isoform X4	0.72	0.589
FAM105A	inactive ubiquitin thioesterase FAM105A	0.902	0.623
HIF1A	hypoxia-inducible factor 1-alpha isoform 1	0.79	0.649
C1orf35	multiple myeloma tumor-associated protein 2	0.81	0.622
PPIL4	peptidyl-prolyl cis-trans isomerase-like 4	0.966	0.612
MELK	maternal embryonic leucine zipper kinase isoform 2	0.756	0.568
C19orf53	leydig cell tumor 10 kDa protein homolog	0.795	0.464
LLPH	protein LLP homolog	0.86	0.637

Supplementary Table S4: Sub-ontology molecular function enrichment of miR-4516 target genes

Enrichment Term	Involved Genes
GO:0046872 metal ion binding	PPP2R3A, PLOD2, RPL37, FSTL1, LUC7L, UBA52, PRKCB
GO:0043169 cation binding	PPP2R3A, PLOD2, RPL37, FSTL1, LUC7L, UBA52, PRKCB
GO:0043167 ion binding	PPP2R3A, PLOD2, RPL37, FSTL1, LUC7L, UBA52, PRKCB
GO:0046914 transition metal ion binding	PLOD2, RPL37, LUC7L, UBA52, PRKCB
GO:0000166 nucleotide binding	U2AF2, PPIL4, MELK, PRKCB
GO:0008270 zinc ion binding	RPL37, LUC7L, UBA52, PRKCB
GO:0046983 protein dimerization activity	HIF1A, POLR1D, CSF1
GO:0003723 RNA binding	U2AF2, PPIL4, RPL37
GO:0005509 calcium ion binding	PPP2R3A, FSTL1, PRKCB
GO:0030528 transcription regulator activity	HIF1A, SUB1, UBA52
GO:0003677 DNA binding	HIF1A, POLR1D, SUB1

Supplementary Table S5: Sub-ontology enrichment terms of biological process (BP)

Term	Genes
GO:0051240 positive regulation of multicellular organismal process	HIF1A, CSF1, UBA52
GO:0045639 positive regulation of myeloid cell differentiation	HIF1A, CSF1
GO:0010647 positive regulation of cell communication	HIF1A, CSF1, UBA52
GO:0016202 regulation of striated muscle tissue development	LUC7L, UBA52
GO:0048634 regulation of muscle development	LUC7L, UBA52
GO:0045637 regulation of myeloid cell differentiation	HIF1A, CSF1

Supplementary Table S6: Sub-ontology enrichment terms of cellular component (CC)

Term	Genes
GO:0022626 cytosolic ribosome	RPL37, UBA52
GO:0033279 ribosomal subunit	RPL37, UBA52
GO:0044445 cytosolic part	RPL37, UBA52

Supplementary Table S7: Increment of miR-4516 expression between baseline and postexposure levels by individuals' characteristics

	good air quality city		
**Variable**	***n***	**miR-4516 (mean, 95% CI)**	***P*-value**
Age, years			
< 40	10	1.43 (−0.96, 3.81)	
40–50	9	1.90 (0.31, 3.48)	
> 50	11	0.48 (−1.44, 2.40)	0.5259
Sex			
Male	15	0.77 (−0.51, 2.04)	
Female	15	1.67 (−0.11, 3.46)	0.3820
